# Grape seed proanthocyanidins supplementation attenuates diquat-induced intestinal barrier damage in weaned pigs

**DOI:** 10.3389/fimmu.2026.1828376

**Published:** 2026-05-07

**Authors:** Yiling Zhang, Xianghong He, Qian Zhao, Heping Li, Rui Qin, Weifang Zuo, Bo Han

**Affiliations:** 1 School of Pharmacy, Chengdu University of Traditional Chinese Medicine, Chengdu, Sichuan, China; 2 CDUTCM-KEELE Joint Health and Medical Sciences Institute, Chengdu University of Traditional Chinese Medicine, Chengdu, Sichuan, China

**Keywords:** Diquat, grape seed proanthocyanidins, intestinal barrier, oxidative stress, weaned pigs

## Abstract

**Objective:**

Grape seed proanthocyanidins (GSP) is a kind of plant polyphenols with a wide variety of biological activities. In the present study, we evaluated whether GSP can alleviate intestinal injury in weaned pigs injected with diquat.

**Methods:**

A total of 32 pigs were randomly assigned to four groups (n = 8): (1) nonchallenged control; (2) control + 50 mg/kg GSP; (3) diquat-treated control and (4) diquat + 50 mg/kg GSP. On the morning of day 15, the pigs were injected with diquat (10 mg per kg body weight) or saline, and were then killed to obtain the serum and intestinal segments 7 days later.

**Results:**

The results showed that GSP addition decreased (*P* < 0.05) the urea concentration and increased (*P* < 0.05) the total superoxide dismutase activity in serum of diquat-challenged pigs. GSP increased (*P* < 0.05) the ileal villus height and duodenal and ileal villus height to crypt depth ratio in diquat-challenged pigs. GSP supplementation not only down-regulated (*P* < 0.05) the cysteinyl aspartic acid-protease-3 (*Caspase-3*), *Caspase-8* and *Caspase-9* expression levels but also up-regulated (*P* < 0.05) the expression levels of *Claudin-1* and *zonula occludens-1* in the small intestine. Furthermore, GSP supplementation increased (*P* < 0.05) the cecal *Lactobacillus*, *Bifidobacterium* and *Bacillus* populations and colonic *Bacillus* population in diquat-challenged pigs.

**Conclusion:**

These findings suggest that GSP can attenuate diquat-induced intestinal mucosa disruption, which was linked to enhancement of the antioxidant capacity and improvement in intestinal microbiota.

## 1 Background

The small intestine not only plays a key role in nutrient absorption but also constitutes the first line of defense against various luminal antigens into sub-epithelial tissues, which primary composed of intestinal epithelial cells and intercellular tight junctions of enterocytes (1, 2), However, a variety of factors, like oxidative stress, characterized by excessive production of reactive oxygen species (ROS), which increased intestinal permeability and facilitates translocation of luminal antigens into sub-epithelial tissues, leading to intestinal damage (3–5). As such, dietary interventions to alleviate the intestinal oxidative stress have attracted considerable research interest worldwide. Accumulating evidence showed that the modulation of the diet can reduce oxidative stress in weaned pigs (6, 7). For instance, dietary supplementation of antioxidant compound attenuates oxidative stress and improves the intestinal function of weaned pigs (8).

Proanthocyanidin is a natural pigment that is abundant in numerous plants and belongs to a kind of flavonoid compound (9). Therein, grape seed proanthocyanidin (GSP), which is a polymeric compound formed by the condensation of phenolic compounds with trihydroxyflavones. Their chemical structure comprises the core anthocyanin structure, wherein a benzene ring is attached to a tricyclic flavonoid scaffold, featuring hydroxyl and methoxy functional groups (10). Importantly, GSP holds various bioactive functions, such as antioxidant (11), anti-inflammatory (12), anti-bacterial (13) and immunomodulatory (14) properties. Importantly, GSP has been utilized as a feed additive in a variety of animal species. For instance, GSP has been indicated to reinforce rumen fermentation function, thereby increasing nutrient digestibility in cattle (15). GSP also can enhance the integrity of the goose intestinal barrier and increase the abundance and diversity of cecal microflora (16). Furthermore, GSP improves growth performance and antioxidant capacity in growing pigs (17).

Although a series of studies revealed a health promoting effect of GSP, evidence establishing direct links among GSP, oxidative stress and intestinal barrier is lacking. Hence, we using diquat that was a classic agent for the construction of oxidative stress to assess the influences of GSP supplementation on oxidative stress-induced intestinal barrier injury in weaned pigs. This study not only sheds light on the mechanisms underlying GSP’s effects on intestinal health in pigs but also facilitate its application in feed industry.

## 2 Materials and methods

All animal procedures performed in the current experiments were approved by the Animal Care and Use Committee of Chengdu University of Traditional Chinese Medicine (Chengdu, China, No. 20240623).

### 2.1 Animals, diet and experimental design

Thirty-two pigs (weaned at 21 day, Duroc × Landrace × Yorkshire) of the average initial body weight (BW) of 6.94 ± 0.26 kg were randomly allotted to four treatments: (1) non-challenged control group (CON): pigs receiving a control diet and injected with NaCl solution; (2) control + GSP (DCON): pigs fed the control diet with supplementation of 50 mg/kg GSP, which was supplied by Guilin Fengpeng Biotechnology Co., Ltd. (Guilin, China) and contains 86.81% proanthocyanidin oligomers, 1.52% catechin, 2.41% epicatechin, and 0.98% proanthocyanidin B2, with a total proanthocyanidin purity of 96.58%; (3) diquat-challenged group (Diquat): piglets receiving the control diet and injected with diquat (Shanghai Aladdin Biochemical Technology Co., Ltd., Shanghai, China); (4) diquat + GSP (DGSP): piglets fed the control diet with supplementation of 50 mg/kg GSP and administration of diquat. The basal diet (**Table 1**) was formulated in accordance with National Research Council (2012) (18). On the 15th day of the trial, the pigs were administered diquat (10 mg/kg of BW) or the same volume saline by intraperitoneal injection. Pigs were raised alone in metabolic cages (1.5 × 0.7 × 1.0 m) at appropriate temperature (26 ± 2 °C) and humidity (60 ± 5%) with *ad libitum* access to water and dies. The feed intake per pig was recorded daily, and pigs were weighed on the mornings of days 15 and 22 of the experiment. The average daily weight gain (ADG), average daily feed intake (ADFI) and the feed conversion ratio (F/G) were calculated.

**Table 1:** Ingredients and nutrient composition of the basal diet (air dry basis, %).

Ingredients	%	Nutrient level	Content
Corn	28.31	Digestible energy (MJ/kg)	14.78
Extruded corn	24.87	Crude protein (%)	19.68
Soybean meal	8.50	Calcium (%)	0.81
Extruded soybean	10.30	Available phosphorus (%)	0.55
Fish meal	4.20	Lysine	1.35
Whey powder	7.00	Methionine	0.42
Soybean protein concentrate	8.00	Methionine + Cysteine	0.60
Soybean oil	2.00	Threonine	0.79
Sucrose	4.00	Tryptophan	0.22
Limestone	0.90		
Dicalcium phosphate	0.50		
NaCl	0.30		
L-Lysine HCl (78%)	0.47		
DL-Methionine	0.15		
L-Threonine (98.5%)	0.13		
Tryptophan (98%)	0.03		
Chloride choline	0.10		
Vitamin premix^*^	0.04		
Mineral premix^†^	0.20		
Total	100		

^*^The vitamin premix provided the following per kg diet: 9000 IU vitamin (V) A; 3000 IU VD_3_; 20.0 IU VE; 3.0 mg VK_3_; 1.5 mg VB_1_; 4.0 mg VB_2_; 3.0 mg VB_6_; 0.2 mg VB_12_; 30.0 mg Niacin; 15.0 mg Pantothenic; 0.75 mg Folic acid; 0.1 mg Biotin.

^†^The mineral premix provided the following per kg diet: 120 mg Fe (FeSO_4_·H_2_O); 20 mg Cu (CuSO_4_·5H_2_O); 15 mg Mn (MnSO_4_·H_2_O); 120 mg Zn (ZnSO_4_·H_2_O); 0.3 mg I (KI); 0.4 mg Se (Na_2_SeO_3_).

### 2.2 Sample collection

At the outset of the trial, feed samples were gathered and preserved for subsequent analysis. During days 11–14 of the trial, fecal samples were collected daily for four consecutive days from each pig to assess nutrient digestibility. Sulfuric acid at a concentration of 10% was added to the fecal samples for nitrogen fixation, and then the samples were dried in an oven at 60 °C for 72 h. The dried samples were then pulverized in a high-speed pulverizer and passed through 0.45 pm filters for chemical assay.

On the 22th day of the trial, blood samples were collected via jugular venipuncture from all pigs after 12-h fasting. The serum samples were obtained by centrifugation at 3,000 × g for 15 min at 4 °C and then stored at −20 °C until analysis. Subsequently, pigs were euthanized with an intravenous injection of sodium pentobarbital (200 mg/kg BW) in accordance with previous studies, and the abdomen was immediately opened to collect the intestinal segments (duodenum, jejunum, and ileum). About 3-cm segments of the middle of duodenum, jejunum and ileum were isolated, gently flushed with ice-cold phosphate-buffered saline (PBS), and then fixed in 4% paraformaldehyde for histological analyses. Afterwards, the mucosae from duodenum, jejunum and ileum were harvested by scraping the segment using a sterile glass slide, snap-frozen in liquid nitrogen, and then stored at −80 °C until analysis. Lastly, digesta from the caecum and colon were collected and stored at −80 °C for measurements of microbial populations and its metabolites.

### 2.3 Apparent nutrient digestibility analysis

Freeze-dried and finely ground feed and fecal samples were analyzed to determine apparent nutrient digestibility using chromium oxide (Cr_2_O_3_) as an indigestible marker. The diet and fecal samples were evaluated for crude ash, crude protein, dry matter and ether extract, according to the AOAC International (2016) (19). An automated oxygen bomb calorimeter (Model 6400, Parr Instrument Co., Moline, IL, USA) was used to evaluate the gross energy (GE) (20). The apparent nutrient digestibility (%) for all parameters was calculated using the following formula: 100 −*A*_1_*F*_2_/*A*_2_*F*_1_× 100, in which A_1_ represents the nutrient content in feces, A_2_ the Cr_2_O_3_ content in feces, F_1_ the Cr_2_O_3_ content in the diet, and F_2_ the nutrient content in the diet.

### 2.4 Serum biochemical analysis

Serum biochemical parameters were measured by automated olympus analyzer (Shanghai, China), including albumin (ALB), alkaline phosphatase (ALP), alanine aminotransferase (ALT), aspartate aminotransferase (AST), globulin (GLB), glucose (GLU), high density lipoprotein cholesterol (HDL-C), low density lipoprotein cholesterol (LDL-C), total cholesterol (TC), triglycerides (TG), total protein (TP) and urea.

### 2.5 Serum antioxidant parameters

Commercial kits from Nanjing Jiancheng Bioengineering Institute (Nanjing, China) were utilized to measure the activities of catalase (CAT), total superoxide dismutase (T-SOD) and total antioxidative capacity (T-AOC) and the concentrations of glutathione (GSH) and malondialdehyde (MDA), according to the manufacturer’s instructions.

### 2.6 Histological characterization

After fixed in 4% paraformaldehyde, the intestinal morphology was estimated. Briefly, the duodenum, jejunum and ileum segments were dehydrated in a graded ethanol series, embedded in paraffin, sliced into 3-µm-thick cross-sections by using a microtome and then stained with hematoxylin and eosin (H&E), which was observed by a Nikon fluorescence microscope (Nikon Corporation, Tokyo, Japan). A minimum of 10 well-orientated crypt−villus units from each intestinal segment were chosen and measured. Villus height and crypt depth were determined using ImagePro Plus 6.0 (Media Cybernetics, Inc., Rockville, MD, USA), and the villus height to crypt depth ratio (V/C) was calculated.

### 2.7 RNA isolation, reverse transcription and real-time quantitative PCR

Total RNA was extracted from the intestinal mucosa by using the RNAiso Plus reagent (Takara Biotechnology Inc., Dalian, China) following the manufacturer’s instructions. The purity and concentration of total RNA were checked by a NanoDrop spectrophotometer (NanoDrop 2000; Thermo Fisher Scientific Inc., Fullerton, CA, USA). Thereafter, the RNA was reverse transcribed into cDNA by PrimeScript RT reagent kit with gDNA Eraser (Takara Biotechnology Inc.). All primers were synthesized by Sangon Biotechnology Co., Ltd. (Shanghai, China) and are presented in **Table 2**. qPCR assays were performed to analysis the mRNA levels of β-actin, B-cell lymphoma-2 (*Bcl-2*), Bcl-2–associated X protein (*Bax*), cysteinyl aspartic acid-protease-3 (*Caspase-3*), *Caspase-8*, *Caspase-9*, *Claudin-1*, *Occludin*, zonula occludens-1 (*ZO-1*), nuclear factor erythroid-derived 2-related factor 2 (*Nrf2*), Kelch-like epichlorohydrin–associated protein 1 (*Keap1*) and heme oxygenase-1 (*HO-1*) by using SYBR Premix Ex Taq II (Tli RNaseH Plus) reagents (Takara Biotechnology Co., Ltd.). Each reaction was performed in a 10 µL reaction volume, which contained 5 µL of SYBR Premix Ex Taq (2×), 1 µL of each primer, 2 µL of doubled-distilled water and 1 µL of cDNA. The procedure of qPCR is as follows: initial denaturation at 95 °C (30 s), followed by 40 cycles between 95 °C (10 s) and 60 °C (25 s), and 72 °C for 5 min. The β-actin gene was chosen as the reference gene, and the target gene mRNA levels were calculated by the 2^−ΔΔCt^ method (21).

**Table 2:** Primer sequences for quantitative real-time polymerase chain reaction.

Gene	Primer sequence (5’-3’)	Product size (bp)	Reference sequence (NCBI)
*β-Actin*	F: TGGAACGGTGAAGGTGACAGC	243	XM_021086047.1
	R: GCTTTTGGGAAGGCAGGGACT		
*Occludin*	R: CTACTCGTCCAACGGGAAAG	237	XM_021086267.1
	F: ACGCCTCCAAGTTACCACTG		
*ZO-1*	R: CAGCCCCCGTACATGGAGA	205	XM_005653786.2
	F: GCGCAGACGGTGTTCATAGTT		
*Claudin-1*	R: CGACTCCTTGCTGAATCTGAACAC	242	NM_001244539.1
	F: CATCTTCTGCACCTCATCATCTTCC		
*Caspase-3*	R: GGGATTGAGACGGACAGTGC	244	XM_021091477.1
	F: TGAACCAGGATCCCGTCCTTTG		
*Caspase-8*	R: TCTGCGGACTGGATGTGATT	248	XM_021080614.1
	F: TCTGAGGTTGCTGGTCACAC		
*Caspase-9*	R: AATGCCGATTTGGCTTACGT	249	XM_021086037.1
	F: CATTTGCTTGGCAGTCAGGTT		
*BAX*	R: AAGCGCATTGGAGATGAACT	256	NM_001301824.1
	F: TGCCGTCAGCAAACATTTC		
*Bcl-2*	R: ATGTGTGTGGAGAGCGTCAA	272	NM_214285.1
	F: GCCCATACAGCTCCACAAAG		
*Nrf2*	F: GCCCAGTCTTCATTGCTCCT	250	XM_005668794.2
	R: AGCTCCTCCCAAACTTGCTC		
*KEAP1*	F: TGTCCTCAACCGTCTGCTCTAC	243	XM_021086794.1
	R: GATCATTCGCCACTCATTCCTCTC		
*HO-1*	F: CGCTCCCGAATGAACAC	199	NM_001004027.1
	R: GCTCCTGCACCTCCTC		

### 2.8 Intestinal microbiological analysis

An estimated 0.1 g intestinal digesta was processed to obtain bacterial DNA with the Stool DNA Kit (Omega Bio-Tek, Doraville, CA, USA) for qPCR, which was performed through the Quant Studio 6 Flex real-time PCR system (Bio-Rad). Primers for total bacteria, *Eescherichia coli*, *Bifidobacterium*, *Lactobacillus* and *Bacillus* are listed in **Table 3**. Absolute quantification was performed using plasmid-derived standard curves to estimate bacterial copy numbers. Amplification was carried out in a 10 μL reaction system containing 5 μL TB Green Premix Ex Taq (2×), 0.5 μL each of forward and reverse primers (100 nM), 1 μL template DNA, and 3 μL nuclease-free water. Thermal cycling was performed with an initial denaturation step at 95 °C for 5 min, followed by 40 amplification cycles consisting of denaturation at 95 °C for 15 s, annealing at 58 °C for 20 s, and extension at 72 °C for 30 s. Amplification specificity was verified by melt curve analysis using the system’s default program. Reaction conditions were optimized to ensure amplification efficiency and specificity, which was confirmed by melt curve profiling.

**Table 3:** Sequences of primers and probes for quantitative real-time polymerase chain reaction.

Item	Primer and probe sequences (5′-3′)	Annealing temperature (°C)	Size (bp)
*Total bacteria*	F: CGGTGAATACGTTCYCGG	55	123
	R: GGWTACCTTGTTACGACTT		
*Bacillus*	F: GCAACGAGCGCAACCCTTGA	55	92
	R: TCATCCCCACCTTCCTCCGGT		
*Bifidobacterium*	F: TCGCGTCYGGTGTGAAAG	55	243
	R: CCACATCCAGCRTCCAC		
*Escherichia coli*	F: CATGCCGCGTGTATGAAGAA	55	101
	R: CGGGTAACGTCAATGAGCAAA		
*Lactobacillus*	F: AGCAGTAGGGAATCTTCCA	55	341
	R: CACCGCTACACATGGAG		

### 2.9 Microbial metabolites analysis

Briefly, the supernatants of approximately 1 g intestinal digesta were centrifuged at 500 × g for 10 min after adding 1:1 distilled H_2_O, 2 mL of supernatant was then transferred to a sterile tube and centrifuged at 12,000 × g for 10 min, after which 1 mL of the supernatant was transferred to a new sterile tube to which 0.2 mL 25% metaphosphoric acid was added. This was left at room temperature for 30 min and then centrifuged at 12,000 × g for 10 min. Next, 500 μL of supernatant was transferred to another sterile tube, to which 500 μL of methanol was added and the mixture, and then was centrifuged at 12,000 × g for 10 min. The supernatant was transferred to a sterile tube and was stored at −20 °C until ready for gas chromatography testing. The short-chain fatty acid (SCFA; acetic acid, propionic acid and butyric acid) were separated and quantified in a gas chromatograph (VARIAN CP-3800, Varian, Palo Alto, CA).

### 2.10 Statistical analysis

Apparent nutrient digestibility data were analyzed by Student’s *t*-test, while other data were analyzed by one-way analysis of variance followed by Tukey’s multiple-range tests by using SAS 9.0 (SAS Institute, Inc., Cary, NC, USA), with each pig as statistical unit. Data are expressed as means and standard errors, and *P* < 0.05 was considered significant.

## 3 Results

### 3.1 Growth performance

As shown in **Table 4**, diquat reduced (*P* < 0.05) the ADFI and ADG of the pigs on days 14−21 and ADFI of the pigs on days 1−21. However, there are no changes (*P* > 0.05) in ADFI, ADG and F/G in diquat-challenged pigs with GSP supplementation.

**Table 4:** Effects of GSP supplementation on the growth performance of weaned pigs challenged with diquat.

Item	Treatment^†^				SEM	*P*-Value		
Days 1-14	CON	GSP	DCON	DGSP		GSP	Diquat	GSP×Diquat
Initial BW, kg	7.00	7.08	6.76	7.00	0.26	0.809	0.779	0.906
Final BW, kg	9.91	9.91	9.01	10.08	0.41	0.563	0.692	0.563
ADFI, g/day	312.23	305.36	298.00	356.43	10.18	0.213	0.366	0.122
ADG, g/day	208.04	202.68	160.71	221.91	16.20	0.688	0.429	0.348
F/G	1.50	1.51	1.85	1.61	0.13	0.558	0.202	0.406
Days 14-21								
Initial BW, kg	9.91	9.91	9.01	10.08	0.41	0.563	0.692	0.563
Final BW, kg	13.1	12.41	10.61	12.05	0.57	0.754	0.248	0.383
ADFI, g/day	695.18^b^	622.68^bc^	478.00^a^	546.67^ac^	27.94	0.962	0.03	0.098
ADG, g/day	455.36^a^	357.14^ab^	228.51^b^	280.95^b^	31.67	0.655	0.011	0.158
F/G	n/a	n/a	n/a	n/a	n/a	n/a	n/a	n/a

Mean values followed by different letters within a row indicate a significant difference (*P* < 0.05).

Data are presented as mean ± standard error (n = 8 pigs/treatment).

^†^CON: pigs were fed with a basal diet; DCON: pigs were fed with a basal diet and intraperitoneally injected with diquat at 10 mg/kg body weight; GSP: pigs were fed with a 50 mg/kg GSP containing diet; DGSP: pigs were fed with a 50 mg/kg GSP containing diet and intraperitoneally injected with diquat at 10 mg/kg body weight.

### 3.2 Nutrient digestibility

The effects of GSP supplementation on apparent digestibility of nutrients are presented in **Table 5**. GSP supplementation increased the apparent digestibility of Ash EE, CP, and GE contrast with the CON group (*P* < 0.05). However, there is no difference (*P* > 0.05) in apparent digestibility of DM between the CON and GSP groups.

**Table 5:** Effects of GSP supplementation on apparent digestibility of nutrients in weaned pigs.

Item	Treatment^†^		SEM	*P*-Value
	CON	GSP		GSP
DM, %	80.55	80.07	0.65	0.724
CA, %	81.51	84.83^*^	0.59	0.002
EE, %	37.11	52.12^*^	2.78	0.004
CP, %	63.31	71.37^*^	1.81	0.020
GE, %	72.67	77.67^*^	1.25	0.045

^*^P < 0.05 versus the CON group.

Data are presented as mean ± standard error (n = 8 pigs/treatment).

^†^CON: pigs were fed with a basal diet; Diquat: pigs were fed with a basal diet and intraperitoneally injected with diquat at 10 mg/kg body weight; GSP: pigs were fed with a 50 mg/kg GSP containing diet; Diquat + GSP: pigs were fed with a 50 mg/kg GSP containing diet and intraperitoneally injected with diquat at 10 mg/kg body weight.

### 3.3 Serum biochemical indicators

**Table 6** reveals that diquat increased (*P* < 0.05) the TC, TG and urea concentrations and decreased (*P* < 0.05) the TP and GLB concentrations in serum. GSP addition decreased (*P* < 0.05) the serum urea concentration and increased (*P* < 0.05) the serum HDL-C concentration in diquat-challenged pigs. Furthermore, either diquat or GSP affect (*P* > 0.05) the serum biochemical indicators, including ALB, ALP, ALT, AST, GLU and LDL-C.

**Table 6:** Effects of dietary GSP supplementation on serum biochemical indices of weaned pigs challenged with diquat.

Item	Treatment^†^				SEM	*P*-Value		
	CON	GSP	DCON	DGSP		GSP	Diquat	GSP×Diquat
ALB (g/L)	22.60	21.25	23.48	21.05	0.73	0.789	0.157	0.673
ALP (U/L)	207.90	222.50	253.87	215.17	11.45	0.627	0.441	0.296
ALT (U/L)	65.09	82.94	65.11	83.01	5.33	0.122	0.996	0.998
AST (U/L)	62.98	52.23	76.12	63.68	5.83	0.117	0.106	0.436
GLB (g/L)	29.71^a^	29.30^ab^	26.78^b^	28.10^ab^	0.41	0.590	0.031	0.318
GLU (mmol/L)	6.70	6.28	6.91	6.16	0.19	0.174	0.907	0.690
HDL-C (mmol/L)	0.95^b^	0.84^ab^	0.76^a^	0.94^b^	0.03	0.528	0.470	0.026
LDL-C (mmol/L)	1.17	1.26	1.28	1.35	0.04	0.402	0.313	0.907
TC (mmol/L)	2.13^bc^	2.01^b^	2.59^a^	2.52^c^	0.09	0.529	0.005	0.881
TG (mmol/L)	0.54^ab^	0.44^b^	0.68^a^	0.56^ab^	0.03	0.088	0.044	0.871
TP (g/L)	52.31^b^	52.78^b^	47.92^a^	49.15^a^	0.70	0.391	0.002	0.698
Urea (mmol/L)	2.16^c^	2.48^bc^	4.57^a^	3.32^b^	0.28	0.132	<0.001	0.024

Mean values followed by different letters within a row indicate a significant difference (*P* < 0.05).

Data are presented as mean ± standard error (n = 8 pigs/treatment).

^†^CON: pigs were fed with a basal diet; DCON: pigs were fed with a basal diet and intraperitoneally injected with diquat at 10 mg/kg body weight; GSP: pigs were fed with a 50 mg/kg GSP containing diet; DGSP: pigs were fed with a 50 mg/kg GSP containing diet and intraperitoneally injected with diquat at 10 mg/kg body weight.

### 3.4 Serum antioxidant capacity

Diquat lowered (*P* < 0.05) the CAT, T-SOD and T-AOC activities and GSH content in serum (**Table 7**). Regardless of diquat, GSP supplementation elevated (*P* < 0.05) the serum T-AOC activity. Importantly, GSP supplementation elevated (*P* < 0.05) the serum T-SOD activity. Additionally, MDA content in serum did not differ (*P* > 0.05) among the four groups.

**Table 7:** Effects of GSP supplementation on serum antioxidant capacity of weaned pigs challenged with diquat.

Item	Treatment^†^				SEM	*P*-Value		
	CON	GSP	DCON	DGSP	SEM	GSP	Diquat	GSP×Diquat
T-AOC (U/mgprot)	2.36^c^	2.78^a^	0.98^b^	1.94^c^	0.20	0.021	0.001	0.317
CAT (U/mgprot)	6.56^bc^	8.98^c^	3.82^a^	3.77^ab^	0.71	0.20	0.001	0.180
MDA (nmol/mgprot)	2.38	2.37	2.96	3.53	0.22	0.062	0.508	0.493
T-SOD (U/mgprot)	63.03^b^	70.55^b^	38.78^a^	66.75^b^	3.96	<0.001	<0.001	<0.001
GSH (mg/mgprot)	2799.07^b^	2855.27^b^	2608.30^a^	2604.36^a^	39.75	0.660	0.003	0.613

Mean values followed by different letters within a row indicate a significant difference (*P* < 0.05).

Data are presented as mean ± standard error (n = 8 pigs/treatment).

^†^CON: pigs were fed with a basal diet; DCON: pigs were fed with a basal diet and intraperitoneally injected with diquat at 10 mg/kg body weight; GSP: pigs were fed with a 50 mg/kg GSP containing diet; DGSP: pigs were fed with a 50 mg/kg GSP containing diet and intraperitoneally injected with diquat at 10 mg/kg body weight.

### 3.5 Intestinal morphology

**Figure 1:**
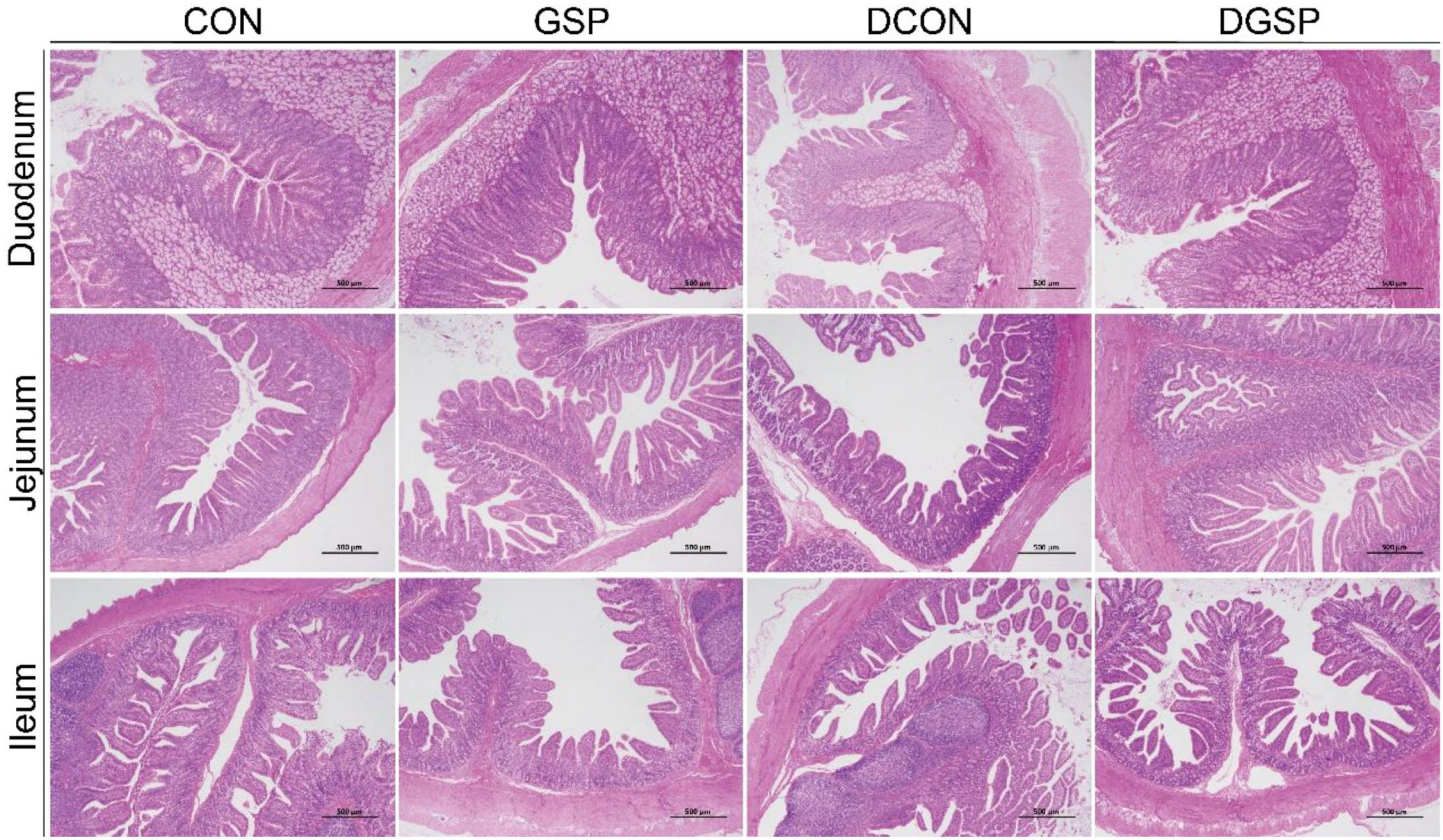
Effects of GSP supplementation on intestinal morphology of weaned pigs challenged with diquat. CON: pigs were fed with a basal diet; DCON: pigs were fed with a basal diet and intraperitoneally injected with diquat at 10 mg/kg body weight; GSP: pigs were fed with a 50 mg/kg GSP containing diet; DGSP: pigs were fed with a 50 mg/kg GSP containing diet and intraperitoneally injected with diquat at 10 mg/kg body weight.

According to the **Table 8**; **Figure 1**, diquat significantly reduced (*P* < 0.05) the duodenal and ileal villus height and duodenal, jejunal and ileal V/C. Irrespective of diquat, GSP supplementation increased (*P* < 0.05) the duodenal villus height and duodenal, jejunal and ileal V/C, decreased (*P* < 0.05) the ileal crypt depth. Importantly, GSP supplementation increased (*P* < 0.05) the ileal villus height and duodenal and ileal V/C in diquat-challenged pigs.

**Table 8:** Effect of GSP supplementation on intestinal morphology of weaned pigs challenged with diquat.

Item	Treatment^†^				SEM	*P*-Value		
	CON	GSP	DCON	DGSP		GSP	Diquat	GSP×Diquat
Duodenum								
Villus height, μm	348.14^b^	366.32^b^	289.47^a^	338.22^b^	9.01	0.008	0.002	0.156
Crypt depth, μm	130.01	135.30	136.10	126.19	5.66	0.696	0.798	0.218
V/C	2.70^b^	2.71^b^	2.13^a^	2.69^b^	0.16	0.030	0.026	0.034
Jejunum								
Villus height, μm	410.26^b^	416.01^b^	311.31^a^	418.26^b^	18.08	0.080	0.123	0.109
Crypt depth, μm	135.52	113.80	122.63	122.11	4.77	0.287	0.820	0.308
V/C	3.03^b^	3.66^a^	2.54^c^	3.43^bc^	0.14	0.020	0.046	0.438
Ileum								
Villus height, μm	356.34^b^	342.04^b^	281.50^a^	336.77^b^	10.00	0.097	0.011	0.015
Crypt depth, μm	125.99^b^	102.64^b^	133.64^a^	103.10^b^	4.54	<0.001	0.381	0.434
V/C	2.83^b^	3.33^c^	2.11^a^	3.29^c^	0.14	<0.001	<0.001	0.012

Mean values followed by different letters within a row indicate a significant difference (*P* < 0.05).

Data are presented as mean ± standard error (n = 8 pigs/treatment).

^†^CON: pigs were fed with a basal diet; DCON: pigs were fed with a basal diet and intraperitoneally injected with diquat at 10 mg/kg body weight; GSP: pigs were fed with a 50 mg/kg GSP containing diet; DGSP: pigs were fed with a 50 mg/kg GSP containing diet and intraperitoneally injected with diquat at 10 mg/kg body weight.

### 3.6 Intestinal barrier function-related gene expression level

**Figure 2:**
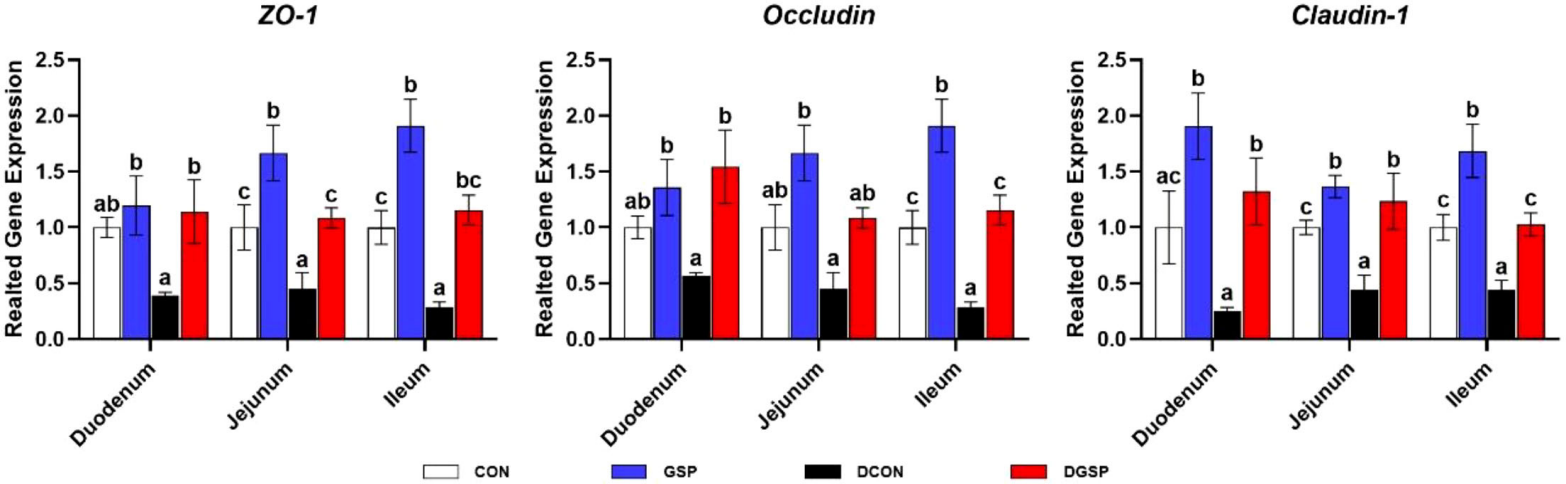
Effects of GSP supplementation on the expression of genes related to intestinal barrier integrity of weaned pigs challenged with diquat. Bars with different letters between groups indicate significant differences (*P* < 0.05). Values are means, with standard errors represented by vertical bars. CON: pigs were fed with a basal diet; DCON: pigs were fed with a basal diet and intraperitoneally injected with diquat at 10 mg/kg body weight; GSP: pigs were fed with a 50 mg/kg GSP containing diet; DGSP: pigs were fed with a 50 mg/kg GSP containing diet and intraperitoneally injected with diquat at 10 mg/kg body weight.

The effects of GSP supplementation on gene expression levels related to intestinal barrier function are presented in **Figure 2**. Compared with the CON group, pigs injected with diquat had lower (*P* < 0.05) expression levels of *Claudin-1* and *ZO-1* in the jejunum and ileum and expression level of *Occludin* in the ileum. Moreover, diquat-challenged pigs supplemented with GSP had higher (*P* < 0.05) expression levels of *Claudin-1* and *ZO-1* in the small intestine and expression level of *Occludin* in the duodenum and ileum.

### 3.7 Intestinal apoptosis-related gene expression level

**Figure 3:**
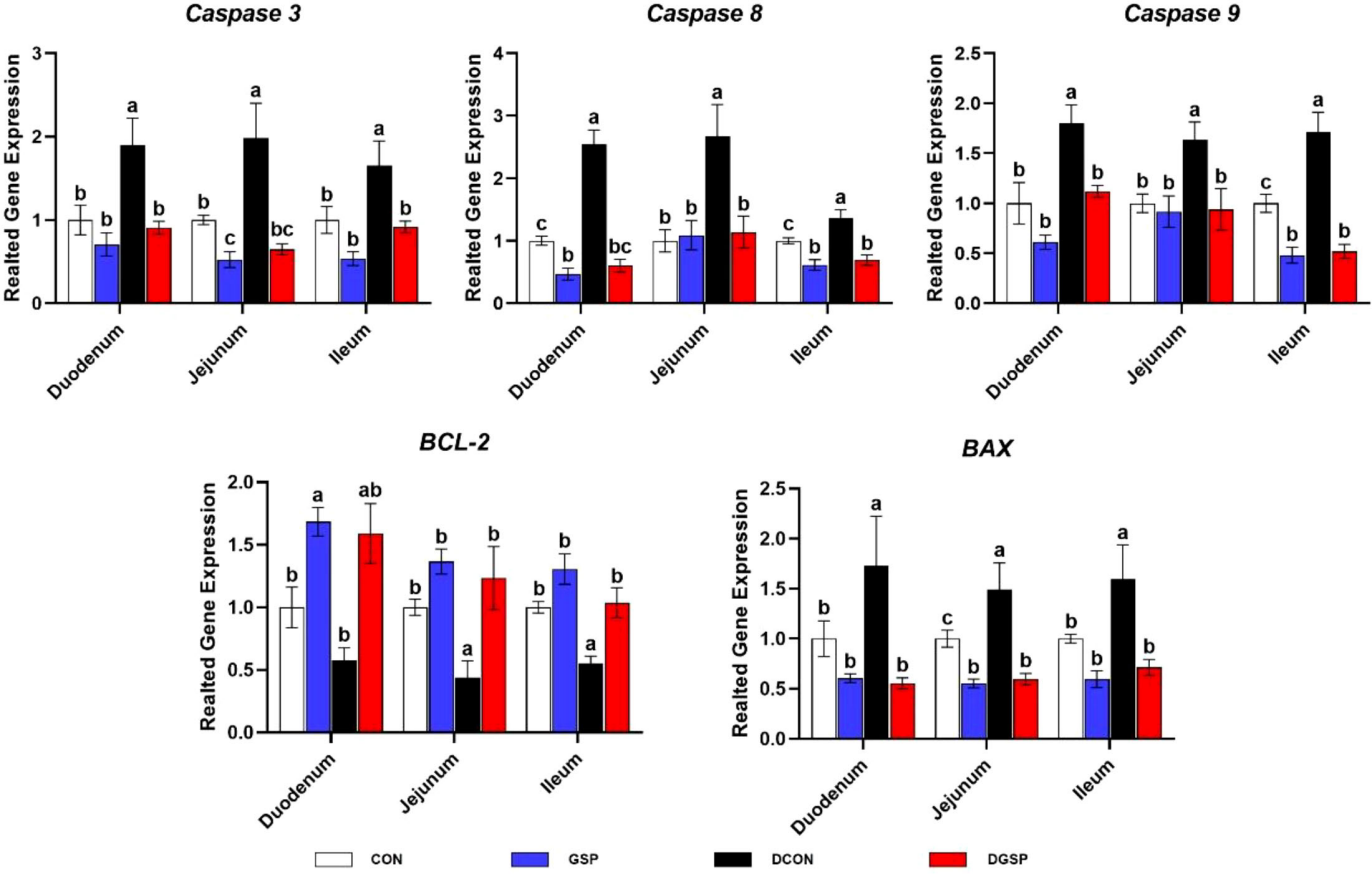
Effects of GSP supplementation on the expression of genes related to intestinal apoptosis of weaned pigs challenged with diquat. Bars with different letters between groups indicate significant differences (*P* < 0.05). Values are means, with standard errors represented by vertical bars. CON: pigs were fed with a basal diet; DCON: pigs were fed with a basal diet and intraperitoneally injected with diquat at 10 mg/kg body weight; GSP: pigs were fed with a 50 mg/kg GSP containing diet; DGSP: pigs were fed with a 50 mg/kg GSP containing diet and intraperitoneally injected with diquat at 10 mg/kg body weight.

As presented in **Figure 3**, diquat challenge up-regulated (*P* < 0.05) the small intestinal *Caspase-3*, *Caspase-8*, *Caspase-9* and *BAX* expression levels, but down-regulated (*P* < 0.05) the small intestinal *BCL-2* expression level. Nevertheless, these gene expression levels (except duodenal *BCL-2*) in diquat-challenged pigs were reversed (*P* < 0.05) by GSP supplementation.

### 3.8 Intestinal antioxidant-related gene expression level

**Figure 4:**
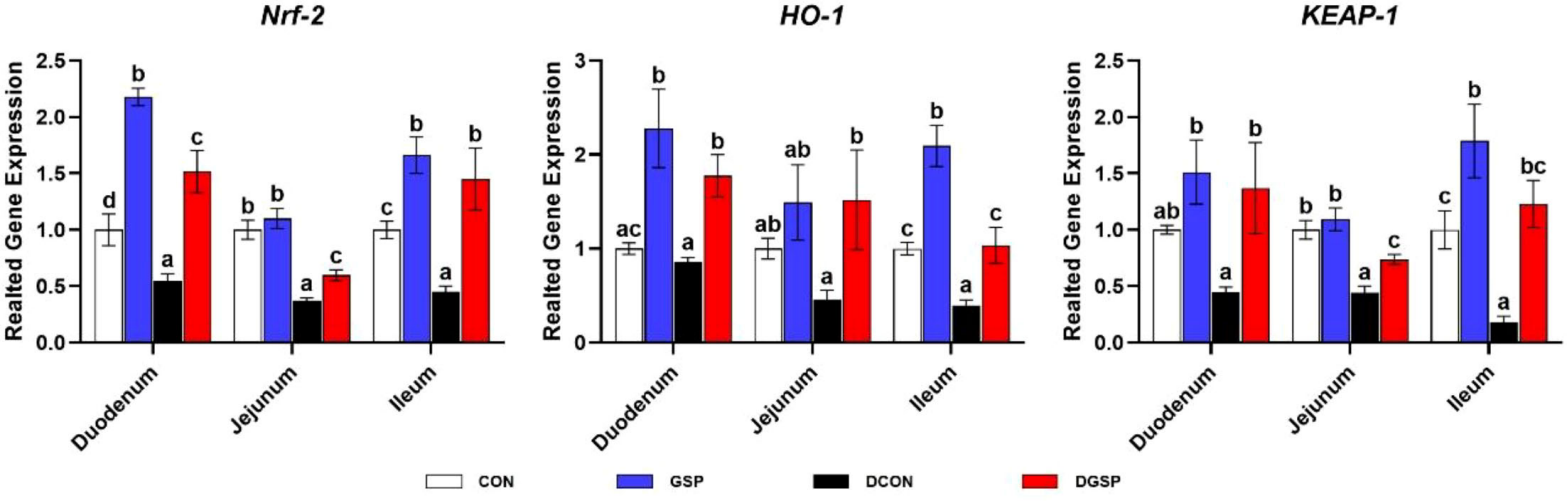
Effects of GSP supplementation on the expression of genes related to intestinal antioxidant capacity of weaned pigs challenged with diquat. Bars with different letters between groups indicate significant differences (*P* < 0.05). Values are means, with standard errors represented by vertical bars. CON: pigs were fed with a basal diet; DCON: pigs were fed with a basal diet and intraperitoneally injected with diquat at 10 mg/kg body weight; GSP: pigs were fed with a 50 mg/kg GSP containing diet; DGSP: pigs were fed with a 50 mg/kg GSP containing diet and intraperitoneally injected with diquat at 10 mg/kg body weight.

**Figure 4** shows diquat injection down-regulated (*P* < 0.05) the duodenal, jejunal and ileal expression levels of *Nrf2*, jejunal and ileal expression levels of *KEAP-1* and the expression level of *HO-1* in the ileum. Nevertheless, GSP supplementation up-regulated (*P* < 0.05) the duodenal, jejunal and ileal expression levels of *Nrf2*, *KEAP-1* and *HO-1* in diquat-challenged pigs.

### 3.9 Intestinal microbial populations and metabolites

**Table 9** indicates that the cecal *Lactobacillus* and *Bacillus* and colonic *Lactobacillus* populations were depressed (*P* < 0.05) after diquat challenge. Meanwhile, acetic acid content in colonic digesta were reduced (*P* < 0.05) after diquat challenge. However, GSP supplementation to diquat-challenged pigs increased (*P* < 0.05) the cecal *Lactobacillus*, *Bifidobacterium* and *Bacillus* populations and colonic *Bacillus* population.

**Table 9:** Effect of GSP supplementation on intestinal microbial populations and metabolites in weaned pigs upon diquat challenge.

Item	Treatment				SEM	*P*-value		
	CON	GSP	DCON	DGSP		GSP	Diquat	GSP×Diquat
Cecal microbial populations, lg (copies/g)								
Total bacteria	11.39	11.22	11.09	11.27	0.06	0.981	0.250	0.120
*Lactobacillus*	4.68^b^	4.69^b^	4.16^a^	4.61^b^	0.07	0.016	0.040	0.020
*Escherichia coli*	6.45	6.59	6.53	6.51	0.21	0.480	0.993	0.385
*Bifidobacterium*	3.88^b^	3.90^b^	3.54^a^	3.95^b^	0.06	0.029	0.101	0.042
*Bacillus*	7.56^b^	7.71^b^	6.46^a^	7.68^b^	0.17	0.006	0.016	0.020
Colonic microbial populations, lg(copies/g)								
Total bacteria	11.66	11.60	11.52	11.54	0.05	0.863	0.317	0.674
*Lactobacillus*	4.68^b^	4.79^b^	4.11^a^	4.67^b^	0.09	0.012	0.011	0.064
*Escherichia coli*	4.34	4.14	4.30	4.11	0.08	0.269	0.843	0.971
*Bifidobacterium*	3.87^ab^	3.81^ab^	3.56^a^	3.98^b^	0.07	0.136	0.558	0.058
*Bacillus*	7.49^b^	7.66^b^	6.21^a^	7.97^b^	0.23	0.040	0.079	0.011
Cecal VFA, mmol/L								
Acetic acid	2951.54^ab^	3442.46^b^	2769.66^a^	3522.44^b^	128.11	0.804	0.014	0.528
Propanoic acid	1317.28	1403.13	1335.20	1203.21	65.31	0.877	0.546	0.473
Butyric acid	676.46^ab^	843.53^a^	622.29^b^	775.04^ab^	37.05	0.033	0.353	0.911
Colonic VFA, mmol/L								
Acetic acid	3598.48	3739.61	2908.27	3851.26	197.00	0.194	0.471	0.325
Propanoic acid	1456.46^ab^	1744.08^a^	1204.19^ab^	2049.16^b^	136.48	0.039	0.911	0.259
Butyric acid	852.12^b^	874.43^b^	390.85^a^	884.02^b^	76.25	0.035	0.057	0.050

Mean values followed by different letters within a row indicate a significant difference (*P* < 0.05).

Data are presented as mean ± standard error (n = 8 pigs/treatment).

CON: pigs were fed with a basal diet; DCON: pigs were fed with a basal diet and intraperitoneally injected with diquat at 10 mg/kg body weight; GSP: pigs were fed with a 50 mg/kg GSP containing diet; DGSP: pigs were fed with a 50 mg/kg GSP containing diet and intraperitoneally injected with diquat at 10 mg/kg body weight.

## 4 Discussion

The small intestinal epithelium is the most important site for nutrient digestion and absorption in pigs (22), while oxidative stress-induced overproduction of ROS was reported to cause the villus–crypt structure disruption (23). At present, we found that GSP exerted a beneficial effect on intestinal morphology, evident by the increased duodenal villus height and duodenal, jejunal and ileal V/C and decreased ileal crypt depth in weaned pigs. Consistently, GSP enhanced the digestibility of CA, EE, CP and GE in weaned pigs. The increased nutrient digestibility may be attributed to the improved intestinal morphology (24). Obviously, diquat prominently reduced duodenal and ileal villus height and duodenal, jejunal and ileal V/C, indicating that a decreased digestion and absorption function of the intestinal epithelium in weaned pigs under oxidative stress (25). However, GSP supplementation relieved the passive influences of diquat on intestinal mucosa, indicating the ameliorate effects of GSP on digestion and absorption function of small intestine in weaned pigs under oxidative stress. These findings combined showed that dietary supplementation with GSP could ameliorate diquat-induced disruption to the intestinal mucosa of weaned pigs.

It is well-known that the intestinal barrier is mainly composed of the intercellular tight junction proteins, such as transmembrane proteins (e.g., occludin and claudin family) and intracellular linker proteins (e.g., ZOs) (26, 27). In the present study, we found that diquat down-regulated the expression levels of *Claudin-1* and *ZO-1* in the jejunum and ileum and expression level of *Occludin* in the ileum. Notably, these gene expression levels in diquat-injected pigs were reversed by GSP supplementation. These results indicated that GSP supplementation could alleviate oxidative stress-induced intestinal barrier dysfunction, which was associated with the improvement of intercellular junctions between epithelial cells. In addition, a previous study suggest that the oxidative stress-induced atrophy of intestinal villus and barrier dysfunction were partially associated with the apoptosis of intestinal epithelial cells (28). In fact, the cell apoptosis is generally regulated by multiple molecules, especially by the Bcl-2 and Caspase families (29). In the present study, diquat injection up-regulated the small intestinal *Caspase-3*, *Caspase-8*, *Caspase-9* and *BAX* expression levels. Importantly, GSP down-regulated these gene expression levels in small intestine. Overall, these results suggest that GSP improved the intestinal barrier integrity by maintaining the tight junction protein expression and suppressing the cell apoptosis in weaned pigs under oxidative stress.

Previous studies revealed that diquat injection could lead to the imbalance of antioxidant status and then cause intestinal barrier impairment (30, 31). GSP is a natural antioxidant, as it has multiple hydroxyl groups in its polyphenolic structure, which can effectively neutralize free radicals and reduce their activity through resonance stabilization (32). Indeed, the antioxidant capacity in diquat-challenged pigs was markedly increased by GSP supplementation, as demonstrated by the elevated serum T-SOD activity. The enhanced antioxidant capacity was also supported by the Nrf2 activation, a master regulator of the antioxidant response, implicating in regulating the expression levels of endogenous antioxidant enzymes that protect against oxidative stress (33). It has been reported that the activities of antioxidant enzymes could be enhanced by upregulating the expression level of Nrf2 (34). Here, we found that GSP up-regulated the duodenal, jejunal and ileal expression levels of *Nrf2* in the diquat-challenged weaned pigs, which was consistent with the increased activity of antioxidant enzyme described above. Taken together, GSP can relieve oxidative stress in weaned pigs by activating the Nrf2 signaling pathway, and thus, increasing the secretion of antioxidant enzymes.

A dynamic but relatively stable intestinal microbiota contributes to intestinal health, while dysbiosis may lead to intestinal damage. Changes in the intestinal microbiota have been shown closely associated with diquat-induced intestinal disruption (35). In the present study, diquat injection inhibited the growth of intestinal beneficial microbiota, such as *Lactobacillus* and *Bacillus*. Importantly, a recent study revealed that dietary supplementation with GSP impacts the intestinal microbiota in growing pigs (36). At present, dietary supplementation with GSP stimulated the growth of health-promoting bacteria, including *Lactobacillus*, *Bifidobacterium* and *Bacillus*, in diquat-challenged pigs. As noted previously, the beneficial microbiota, like *Lactobacillus*, could affect intestinal barrier integrity by regulating the gene expression level of tight junction proteins (37, 38). Therefore, GSP supplementation led to intestinal microbiota alternations contribute to repair diquat-induced intestinal barrier injury in weaned pigs.

## 5 Conclusions

In summary, the present study demonstrated that GSP supplementation exerts beneficial effects in ameliorating diquat-induced oxidative damage in weaned pigs. The improvement in intestinal health by GSP is likely associated with elevated antioxidant capacity and modified intestinal microbiota. This study not only indicated a beneficial effect of GSP supplementation on pigs but also contributed to understanding the mechanisms behind the GSP-regulated biological functions.

## Data Availability

The original contributions presented in the study are included in the article/supplementary material. Further inquiries can be directed to the corresponding author.
